# Dismembered porcine limbs as a proxy for postmortem muscle protein degradation

**DOI:** 10.1007/s00414-021-02571-6

**Published:** 2021-05-06

**Authors:** J. Geissenberger, B. Ehrenfellner, F. C. Monticelli, Stefan Pittner, Peter Steinbacher

**Affiliations:** 1 Department of Biosciences, University of Salzburg, Hellbrunner Str. 34, 5020 Salzburg, Austria; 2 Department of Forensic Medicine and Forensic Neuropsychiatry, University of Salzburg, Salzburg, Austria

**Keywords:** Protein, PMI estimation, Degradation, Muscle, Pig

## Abstract

The estimation of the postmortem interval (PMI) is of critical importance in forensic routine. The most frequently applied methods, however, are all restricted to specific time periods or must be excluded under certain circumstances. In the last years it has been shown that the analysis of muscle protein degradation has the potential to contribute to according delimitations in practice. In particular, upon biochemical analysis, the specific time points of degradation events provide reasonable markers for PMI delimitation. Nevertheless, considerable research is yet required to increase the understanding of protein decomposition and how it is affected by individual and environmental influencing factors. This is best investigated under standardized conditions, however, a considerate selection of proxies, regarding costs, effort, and expected outcome is required. Here, we use pigs to compare muscle protein decomposition in whole bodies and dismembered body parts (amputated hind limbs). Not only do experiments on body parts reduce the costs and allow easier handling in basic research, but also they aid to investigate the practical application of PMI estimation in dismembered body parts, or other extensive injuries, which are not unusual scenarios in crime investigation. Specifically, we investigated whether there are differences in the degradation rates of selected muscle proteins, sampled from dismembered legs and from hind limbs attached to whole pig bodies. Our results show distinct time-dependent degradation patterns of muscle proteins in a predictable manner regardless of sample origin. We are able to demonstrate that amputated hind limbs are suitable proxies for the analysis of muscle protein degradation, especially to investigate certain influencing factors and establish according standardized models.

**Supplementary Information:**

The online version contains supplementary material available at 10.1007/s00414-021-02571-6.

## Introduction

A most precise determination of time since death plays a major role in forensic medicine. It is of critical importance to define the postmortem interval (PMI) as precisely as possible in order to substantiate witness testimonies or to exclude or incriminate suspects in a crime investigation. The most precise and prominently used approach is the temperature method, which is based on temperature measurements of a dead body and the environment and its association to a postmortem temperature decrease model [1]. This method is only applicable in the first 36–48 h postmortem until the body core temperature has adapted to the environment [2]. Within the same time frame, PMI delimitation is also possible by examination of supravital reactions of electrically stimulated facial muscles [3], and the development of rigor and livor mortis [4]. These phenomena, however, are as well restricted to specific time frames [5] and certain circumstances surrounding death [6]. In advanced phases of decomposition, forensic entomology can be used for PMI determination [7, 8]. Despite being the most reliable source for PMI estimation in advanced stages postmortem, forensic entomology also has its limitations due to several parameters that can prevent or delay insect colonization (weather, burial of the corpse, low temperature etc.) [7].

An innovative approach for PMI estimation based on postmortem degradation of skeletal muscle proteins has been proposed recently [9]. The high potential of this approach arises from the fact that postmortem degradation patterns of some of these proteins were predictable and reproducible in different species [9–11] including humans [12]. In addition, the biochemical analysis of muscle protein is methodologically simple and can provide results in a short amount of time. Thus, the analysis of proteins is a promising new method to the methodological forensic tool kit, especially for mid-term and late PMI estimation. However, there are some factors that can influence protein degradation processes, for instance ambient temperature and humidity, as well as individual factors like body weight and age [12]. This has to be taken into consideration when establishing novel methods. It is also important to use suitable experimental models in order to create standardized protocols for analyses of postmortem changes. Since experiments on humans are virtually impossible to standardize due to the variety of internal and external influencing factors, animals are often the preferred option. Especially, pigs have proven to be the best choice for human proxies in forensic research purposes [13], not only due to easy access to these animals, but also due to their similarity to humans regarding size, body composition, skin coverage with hair, gut microbiota, and more [13]. Since experiments on whole pigs are associated with elaborate logistical effort and considerably larger expenses, studies based on amputated body parts and/or explanted organs and tissues are often preferred [13–15]. However, only very few studies compared the degradation processes in whole bodies and amputated body parts. In this regard, it has been shown that the decomposition rate in dismembered body parts is slower than in whole bodies, as the microbiota in gastrointestinal organs increase body temperature and thus enhance the putrefaction process via anaerobic fermentative processes [16]. In addition, the lower mass of dismembered body parts compared with whole bodies leads to a faster cooling and thus decreased decomposition velocity of such body parts [17, 18]. To date, no information exists about possible differences of protein degradation rates in dismembered body parts and whole animals as all previous studies used either whole pigs or pig legs [9, 19–21]. Despite providing detailed information about protein degradation among others, the results of these studies can hardly be compared. Thus, additional data on differences in decomposition processes of whole bodies and dismembered body parts is required as such knowledge can also be of interest for forensic cases in which dismembered bodies are involved.

The present study addresses these issues by analyzing postmortem protein degradation in both pig muscles from dismembered hind limbs as well as from legs attached to whole pig bodies. In addition, a new set of proteins is tested in order to eventually add markers for PMI estimation, with a focus on advanced PMI changes. Proteins examined in this study were chosen from previous meat science studies which investigated storage conditions and/or tenderness of meat (alpha-actinin, alpha-tubulin, tropomyosin, and vinculin) [22–24], as well as from a recent proteomic study that specifically looked for suitable biomarkers for PMI estimation (GAPDH) [25]. In particular, these proteins were found to be especially good candidates to estimate the PMI in mid-term and/or late phases. Western blot analysis was employed to examine the degradation behavior of these proteins in whole pigs and pig legs that were stored at room temperature for 10 days under standardized conditions. Knowing about differences in the decomposition of body parts versus whole bodies has most relevant implications for the applicability of body parts as models for forensic science as well as for routine work when only body parts are available for investigation.

## Materials and methods

### Animals and muscle sampling

Six sub-adult pigs (1 male, 5 female, commercial crossbreed animals, German Large White × German Landrace, 5 months old, 50 ± 3 kg) were used for this study. The animals were killed in a slaughterhouse according to standard procedures. Both hind limbs from three pigs (1 male, 2 female) were dissected immediately after death. Together with the three remaining whole animals, the dismembered legs were transported to the lab and stored in a climate chamber under constant conditions (temperature 20 ± 2 °C; humidity 50 ± 5% rH). Muscle samples were collected at 18 pre-defined time points after death, respectively, at 0, 6, 12, 18, 24, 36, 48, 60, 72, 84, 96, 108, 120, 144, 168, 192, 216, 240 h postmortem (hpm). The first samples were taken directly (approximately 5–10 min) after death at the slaughterhouse (= 0 h reference samples). For each sampling, an incision was made through the skin and the underlying fascial layer, using a surgical scalpel, and muscle samples (approx. 5 × 5 × 5 mm) of the *M. biceps femoris* were taken via biopsy. Samples were collected from 2 cm depth and a minimum distance of 2 cm was kept between different sampling sites. Muscle samples were snap frozen and stored in liquid nitrogen until further processing.

### Sample processing

Cryogenic grinding and subsequent sonication via ultrasound (2 × 100 Ws/sample) were performed in order to homogenize the samples. For processing, 10 × *v*/*w* RIPA buffer containing protease inhibitor cocktail (SIGMA) was used as lysis and protein extraction buffer to prevent further muscle protein degradation. Homogenized sample solutions were centrifuged at 1000×*g* for 10 min and supernatant was transferred and stored at − 20 °C until further use. Protein concentrations were measured by using Pierce BCA-Assay Kit (Thermo Fisher Scientific Inc.).

### SDS-PAGE

All samples were diluted with double distilled water to specific overall protein content (30 μg for vinculin and alpha-tubulin, 15 μg for α-actinin and tropomyosin, 10 μg for GAPDH) prior to analysis. Electrophoresis was performed according to Laemmli with some adaptations [26] and run on 10% polyacrylamide resolving gels (acrylamide/N,N′-bis methylene acrylamide = 37.5:1, 0.1% SDS, 0.05% TEMED, 0.05% APS, 375 mM Tris HCl, pH 8.8) and 5% stacking gels (acrylamide/N,N′-bis methylene acrylamide = 37.5:1, 0.1% SDS, 0.125% TEMED, 0.075% APS, 125 mM Tris HCl, pH 6.8). Sample dilutions were denatured at 90 °C for 5 min prior to insertion into the stacking gel wells. Electrophoresis was run at a constant voltage of 150 V until the dye front reached the bottom of the resolving gel (approximately 2 h). The running buffer contained 25 mM Tris pH 8.3, 195 mM glycine, 2 mM EDTA, and 0.1% SDS. Following electrophoresis, proteins were transferred from the gels onto polyvinylidene fluoride (PVDF) membranes in transfer buffer (containing 192 mM glycine, 20% methanol, and 25 mM Tris pH 8.3). Transfer (electroblotting) was run at a constant current of 250 mA for 75 min. Membranes were then stored at − 20 °C until further use.

### Western blotting

All membranes were blocked for 1 h in a blocking buffer containing PBST (137 mM NaCl, 10 mM Na_2_HPO_4_ anhydrous, 2.7 mM KCl, 1,8 mM KH_2_PO_4_, 0.05% Tween) and 1% bovine serum albumin as blocking agent (BSA; albumin bovine fraction V, pH 7.0). The following primary antisera were used: mouse-monoclonal anti-vinculin (7F9, Santa Cruz Biotechnology, 1:1000), mouse monoclonal anti-α-actinin (H-2, Santa Cruz Biotechnology, 1:1000), mouse monoclonal anti-tropomyosin (CH1-s, DSHB, 1:500), mouse monoclonal anti-α-tubulin (12G10, DSHB, 1:500), mouse monoclonal anti-GAPDH (6C5, Santa Cruz Biotechnology, 1:1500). HRP-conjugated polyclonal goat anti-mouse immunoglobulins (Dako, 1:10^.^000) were applied as secondary antibodies. All primary and secondary antibodies were diluted in blocking buffer and incubated for 1 h. After each antibody application, membranes were extensively rinsed and washed (3 × 10 min) in PBST. Visualization of antibody binding was enabled by application of chemiluminescence substrate (Roti®-Lumin plus, Carl Roth) and photographed using a digital gel documentation system (Fusion X, Vilber).

### Statistical analysis

The intensity of all protein bands was measured using the gel analysis tool of ImageJ software (v.1.48 NIH, National Institutes of Health, USA). Histograms of the tonal distribution of the images were plotted and the area underneath the graphs was measured according to the program’s standard protocol. Band patterns of the 0 hpm samples were used as control and considered the native form of the protein. All band signals on the blot with ≥ 1% relative density (compared with respective dominant control band) were considered a present protein band, all signals < 1% of the respective control band were considered background. This enabled a binarization of the results and provided information on the absence (0) or presence (1) of proteins and degradation products. The abundance of bands per time point was statistically analyzed and logistic regressions were calculated for all significant correlations of protein changes with a significance level above 0.95. This represents the changing presence probability of a protein band (native form and/or degradation products) over the PMI, and thus allows a prediction of the time since death at which the presence of a specific degradation product can be expected in a significant number of cases (*P* > 95 %), when a protein is degraded (*P* < 5%), or when a change is more likely to have occurred than not (*P* = 50%). All statistical analysis was performed using SPSS Statistics 26 (IBM, USA).

## Results

**Fig. 1:**
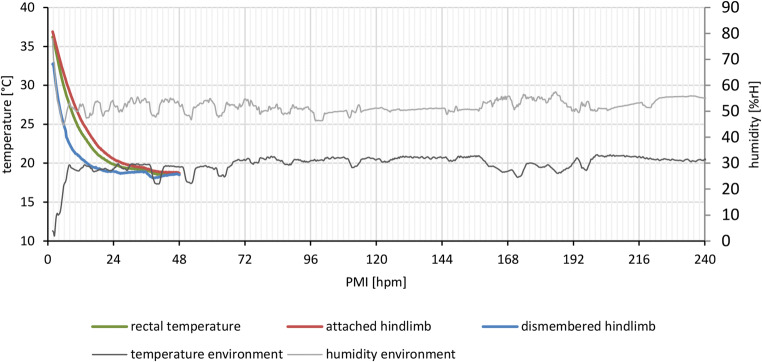
Postmortem temperature adjustment of a whole pig carcass (rectal and hind limb temperature), as well as from an amputated hind limb, in dependence of environmental temperature and humidity. Temperature data from pigs were measured for the first 48 hpm until equilibrium was reached. Environmental conditions were measured throughout the entire time course of 240 hpm

Data of temperature measurements revealed that the dismembered limbs cooled faster in comparison with the attached legs and the body as measured in the rectum. An equilibrium with environmental temperature was reached after approximately 12–14 h in dismembered legs and after 24–30 h in attached legs and rectum (Fig. 1). Muscle and rectal temperature measurements were terminated after 48 hpm, while data collection of ambient temperature and humidity proceeded throughout the whole experiment.

In general, results of investigated muscle proteins provided predictable degradation patterns, in similar matter regardless of sample origin (attached or dismembered). The data showed that vinculin, GAPDH, and alpha-actinin break down into fragments of smaller molecular weight (degradation products) during different stages of postmortem degradation. By contrast, alpha tubulin degraded without detectable split products, while tropomyosin remained stable and maintained the native protein band pattern over the investigated time course of 10 days.

**Fig. 2:**
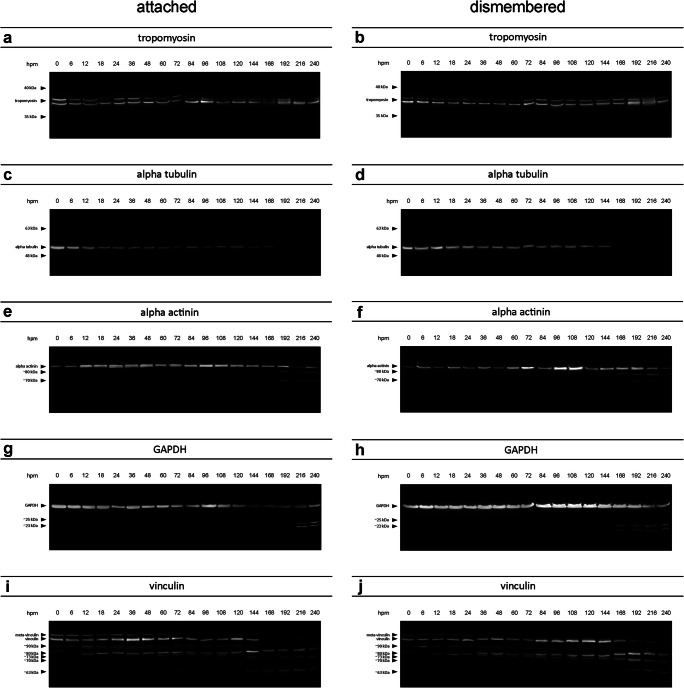
Representative Western blot results, depicting degradation patterns of tropomyosin, alpha-tubulin, alpha-actinin, GAPDH, and vinculin of pigs exemplary for attached (**a**, **c**, **e**, **g**, **i**) and dismembered (**b**, **d**, **f**, **h**, **j**) hind limb muscle samples over the time course of 240 hpm

In detail, tropomyosin appeared as a double band at approximately 36 and 38 kDa representing two isoforms of the protein. In all analyzed samples in both attached and dismembered hind limbs, these two bands remain present in this characteristic state over the investigated time course (Fig. 2a, b). No degradation products were detectable in any of the samples regardless of time point and/or condition.

**Fig. 3:**
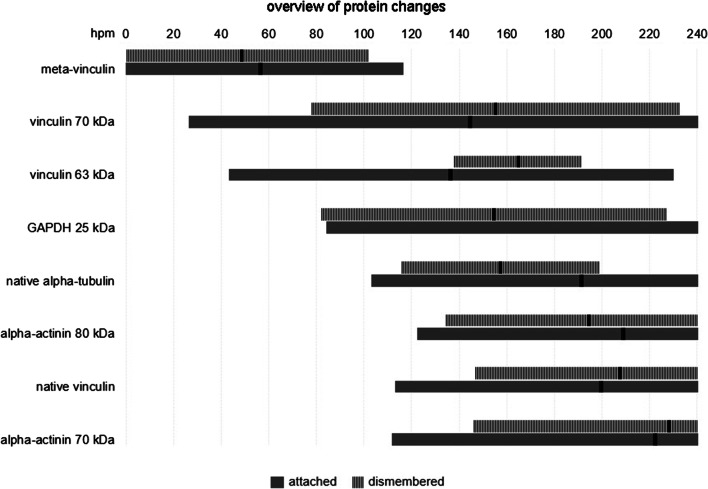
Timeframes of protein degradation events. Gray bars indicate the temporal range in which a degradation event is most likely to happen (between 5 and 95 %), as well as *P* = 50% values (bold black lines), indicating the time-points after which a degradation event has more likely already occurred than not

Alpha-tubulin depicted a single native protein band with a molecular weight of about 49 kDa. Similar to tropomyosin, it remained stable without the formation of any degradation products until 120 hpm in both attached and non-attached hind limb samples (Fig. 2c, d). Afterwards, a complete degradation of alpha-tubulin was detected in five amputated pig legs from 169 hpm onwards (Fig. 2c) and in all non-amputated samples from 216 hpm onwards (Fig. 2d). Logistic regression analysis revealed that *P* = 50% values of alpha-tubulin degradation are reached at 190.7 hpm in attached and after 156.9 hpm in dismembered hind limbs (Fig. 3). Furthermore, native alpha-tubulin is significantly present until 278.7 hpm in non-amputated hind limbs and until 197.9 hpm in amputated hind limbs (> 95% likelihood of band presence) (Fig. 3). For detail, see Supplementary material Fig. S1e.

Analysis of alpha-actinin showed a single native band with a molecular weight of about 100 kDa (Fig. 2e, f). Only two samples from dismembered hind limbs showed a loss of the native band at 240 hpm. Additional bands (degradation products) were detectable in late PMI phases (≥ 168 hpm) in both amputated and non-amputated hind limbs. Five attached hind limbs displayed degradation products of approximately 80 kDa and 70 kDa with high regularity from 192 hpm onward (Fig. 2e). In only one leg, a third split product appeared after about 84 hpm with a molecular weight of approximately 60 kDa. Logistic regression analysis revealed that the degradation product with a molecular weight of 80 kDa was significantly present only after 294.4 hpm in attached hind limbs and after 254.4 hpm onwards in all samples collected from amputated hind limbs. Both of these values are outside the investigated timeframe and thus disregarded from interpretation (Fig. 3, Fig. S1f). In 4 of 6 cases, a second band of approximately 70 kDa appeared from 168 hpm onward (Fig. 2f). As in non-amputated pig legs, only one of the dismembered hind limbs showed a third degradation product at approximately 60 kDa in late time points postmortem (216 hpm onwards). *P* = 50 % values of the 80 kDa alpha-actinin band were statistically reached at 208.3 hpm in attached and 194.0 hpm in dismembered hind limbs, respectively. The occurrence of 70 kDa alpha-actinin was more likely than not at 221.8 hpm in attached, and at 227.5 hpm in non-attached hind limbs (Fig. 3).

Similarly, analysis of GAPDH Western blots revealed a stable native protein band with a molecular weight of approximately 37 kDa over the investigated time period in both amputated and non-amputated hind limbs (Fig. 2g, h). However, half of the muscle samples collected from non-amputated hind limbs showed additional protein bands in advanced time points postmortem (Figs. 2g and 3, Fig. S1d). These degradation products consisted of protein bands with molecular weights of 25 kDa and 23 kDa and occurred at the same time when present. In a similar fashion, the same degradation products appeared in the muscle samples from amputated hind limbs. Again, they were detectable predominantly in late PMI phases (from 168 hpm onward). In one leg, they occurred earlier (from 84 hpm onward), but logistic regression analysis revealed that especially the 25 kDa split product was significantly present from 226.2 hpm in dismembered hind limbs (Figs. 2g and 3, Fig. S1d). Statistical analysis revealed that *P* = 50% value of GAPDH 25 kDa is reached at 154.0 hpm in attached hind limbs (Fig. 3). In dismembered hind limbs, the calculated value exceeded the sampled timeframe.

Western blot analysis of vinculin showed similar degradation patterns in amputated and non-amputated hind limbs with multiple degradation products even in early postmortem stages (Fig. 2i, j). All examined hind limbs depicted the native 117 kDa vinculin band (Fig. 3, Fig. S1g). Although significance levels again exceeded the sampled timeframe, the *P* = 50% values of native vinculin were reached at 199.2 hpm in attached, and at 207.1 hpm in dismembered hind limbs (Fig. 3). An additional protein band at approximately 135 kDa, referred to as the splice variant meta-vinculin [24, 25], degraded until 72 hpm in both models. Only one attached hind limb displayed a complete lack of the vinculin splice variant. Logistic regression analysis showed that meta-vinculin was significantly present until 115.5 hpm and reached its *P* = 50% value at 56 hpm in attached hind limbs. Dismembered hind limbs showed a presence of meta-vinculin until 101 hpm and reached the *P* = 50% value at 48.2 hpm (Fig. 3, Fig. S1a). All investigated samples exhibited additional protein bands at approx. 90 kDa and 80 kDa. The split product of 80 kDa remained stable over the investigated time course, whereas the band intensity of 90 kDa degradation product decreased with increasing PMI (from 168 hpm onward), with only one exception (one dismembered hind limb). Here, the split product occurred in all samples between 24 and 240 hpm. Another band at about 73 kDa occurred in all tested legs between 12 and 144 hpm. Additional bands with molecular weights of approx. 70 kDa, 67 kDa, and 63 kDa were also displayed in both amputated and non-amputated hind limb muscle samples. A single non-amputated sample exhibited these degradation products from 36 hpm onward; in all others, they occurred only in later PMI phases (from 144 hpm onwards). In amputated hind limb samples, appearance of these split products was shown at slightly later phases postmortem (from 168 hpm onward). The 67 kDa protein band was only detectable in three attached and in two amputated hind limbs. Logistic regression analysis showed that the 70 kDa vinculin band is significantly present from 231.4 hpm onwards in amputated hind limbs, in non-amputated hind limbs; however, the statistical value lied beyond the sampling frame (261.6 hpm) (Fig. 3, Fig. S1b). The 63 kDa vinculin band exceeded the 95% confidence limit at 229 hpm in samples from non-amputated and at 190.6 hpm in amputated legs (Fig. 3, Fig. S1c). Statistics revealed that *P* = 50% values of 70 kDa vinculin are reached at 144.1 hpm in attached and 154.9 hpm in dismembered hind limbs. *P* = 50 % values of 63 kDa vinculin were 135.9 hpm in attached and 164.1 hpm in dismembered hind limbs (Fig. 3)

Figure 3 depicts a summary of proteins and/or degradation products significantly changing band quality during the investigated time period.

## Discussion

This study focused on postmortem protein degradation in attached or dismembered hind limbs in order to find an optimal model for future research in time since death estimation. With this comparative study, we were able to demonstrate that body parts of animals (in this case dismembered pig legs) are suitable models for investigation of decomposition processes in skeletal muscles because proteins largely degrade in a similar fashion regardless of origin (amputated or non-amputated hind limbs). The degradation patterns of investigated muscle proteins occurred in a predictable manner and with a discrete dependence upon time since death. In resemblance to previous studies, proteins degraded in early (meta-vinculin), but especially advanced PMI stages (alpha-tubulin, alpha-actinin, GAPDH, vinculin), or remained stable (tropomyosin) over the investigated time course (10 days postmortem). Furthermore, the time-dependent decrease and loss of the native proteins were, in parts, accompanied by the formation of split products with a high consistency at distinct time points postmortem. Thus, again, the analysis of the degradation patterns of skeletal muscle proteins is confirmed to be a very promising approach for PMI delimitation in the first 10 days post mortem [see review of Zissler et al. 2020] [27]. However, it should be noted that also proteins of other tissues have potential to aid in PMI delimitation, from very early [28, 29] to late PMI phases [30, 31].

Results suggest that—despite qualitative and chronological resemblance in protein degradation patterns between amputated and non-amputated hind limbs—muscle samples from dissected hind limbs provide even more predictable (and definable) results and protein changes occur within a shorter time range compared with samples obtained from attached hind limbs (Fig. 3). Statistical analyses and logistic regression curves revealed that in dismembered hind limbs, muscle proteins (e.g., vinculin, alpha-tubulin) and degradation products (e.g., 70 und 63 kDa vinculin, 80 kDa alpha-actinin, 25 kDa GAPDH) degrade with high regularity at specific time points (Fig. 3, Supplements Fig. S1). All logistic regression curves of muscle proteins in dismembered hind limbs are steeper than in attached hind limbs, demonstrating that protein changes occur within a narrower time frame. This allows—with a combination of different proteins and degradation events in specific time frames—to develop a standardized PMI model and to investigate specific influencing factors better by using dismembered pig legs. In this context, *P* = 50% values are also valuable indicators to assess differences in the progress of degradation. They show the time points when protein changes are more likely to occur than not. With consideration of the faster cooling of dismembered hind limbs (Fig. 1), this suggests that proteins in attached hind limbs reach this time point faster than in dismembered hind limbs. However, results show that some degradation events occurred slightly earlier, and others slightly later in attached and dismembered hind limbs, respectively, and much rather lie within a certain range of variance. In addition, in detached legs, the changes occurred within a narrower time frame, suggesting this as a preferable proxy for the basic analysis of protein decomposition and its influencing factors.

Nevertheless, when transferring these results onto whole pig bodies, or even humans, adaptations have to be made. Due to interspecies differences between pigs and humans [13, 32, 33], as well as individual variabilities within humans [34], several influencing factors have to be taken into consideration when adapting standardized models for practical routine work. Despite strong evidence that postmortem protein degradation kinetics and protein alterations are similar across various mammalian species [11, 19, 33–37], the generalizability of human decomposition is of primary interest for forensic routine casework. A high number of human cases and a database providing individual details and environmental circumstances before death are recommended in order to get a reliable outcome and better comparison between standardized animal models and realistic forensic cases. In this context, it is also advised to expand this study by investigating protein degradation at different ambient temperatures and/or other standardized environmental conditions in order to get a reasonable model for protein degradation patterns depending on PMI and temperature (among others).

In contrast to other investigated proteins in this study, tropomyosin remained unchanged and its double band native form was unaffected by degradation processes over the observed time period and with no regards to different sample origin (attached or amputated). Therefore, and in agreement with previous findings [9], it can be considered to be a reliable positive control within the first 10 days postmortem. However, since tropomyosin is a known substrate for calpain cleavage [38], the protein might undergo degradation in advanced PMI phases.

Vinculin and its degradation products proved to be promising markers to delimitate time since death. Meta-vinculin was detectable only in early stages after death and vanished within the first 3 days postmortem with a high regularity in both amputated and non-amputated hind limbs. These results show a resemblance to previous studies where this splice variant degraded with increasing PMI in pigs [19] and other species including humans [10, 35]. Appearance of these degradation products was predictable and occurred in the same manner in both, attached and dismembered hind limbs. Similar findings were obtained in pigs [19] and chicken [11], where the intact protein was not detectable after 4 days postmortem. Despite slight differences between amputated and non-amputated hind limbs, the chronological order of the native band loss, accompanied by the appearance of (transient) split products, was alike.

Similarities in protein degradation behavior between attached and dismembered hind limbs were also found by analysis of the proteins GAPDH, alpha-actinin, and alpha-tubulin. The loss of the native band (alpha-tubulin) and the detection of degradation products (GAPDH, alpha-actinin) occurred in a similar manner and particularly in late PMIs. These findings confirm results of previous studies with different species such as pigs [36], mice [10], rats [37, 39], geese and ducks [40], and also humans [10, 25].

In summary, the present study has shown no major qualitative difference in postmortem protein degradation between muscle samples from whole model animals and dissected hind limbs. We were able to identify several proteins with similar degradation behavior in both amputated and non-amputated pig legs. These degradation patterns show close resemblance to results from previous studies with several other vertebrates and in parts also humans. From the results, it can be inferred that amputated pig legs are able to serve as a suitable proxy to investigate influencing factors on postmortem protein degradation to delimitate time since death. In addition, the study provides new suitable protein markers for PMI estimation and their degradation behavior at room temperature under standardized conditions.

It is important to establish suitable experimental models to advance knowledge about forensic research questions and postmortem protein degradation (for time since death delimitation) in particular. In this context, animal models are best suited for methodological proof of principle, the analysis of influencing factors under standardized conditions, as well as the detection of new PMI markers. Pig cadavers as model animals have proven to be the best practical choice for forensic research purposes [13]. Depending on the specific question, the use of pig carcasses and/or porcine tissue to study human taphonomic processes is reasonable, not only due to easy replication at low expenses [14], better controlling mechanisms owing to restricted influencing factors (internal and environmental) and availability of appropriate control samples, but also because the access to animals for scientific research is much easier [13, 15]. Although there are overlapping observations regarding insect colonization [41, 42], similar morphological changes during decomposition [43], and interspecies similarities in postmortem protein degradation between humans and animals [10], animal models provide only limited progress for routine application and humans are the preferred subject for forensic research [44] and experiments based on human corpses are necessary for final validation of forensic methods and obtained data from animal models. However, in order to investigate novel aspects (new biomarkers and/or influencing factors), it is reasonable to resort to standardized animal models.

Knowledge of degradation processes in dismembered body parts can as well be of interest in crime investigation, since it is a plausible scenario to be confronted with. With current methods, PMI estimation is extremely difficult if not impossible when only body parts are available or dismembered corpses are found. The present study demonstrates that differences in protein degradation based on sample origin (attached or dismembered hind limbs) can be largely ruled out. This confirms findings of previous investigations that, when certain environmental factors such as differences in oxygen levels, humidity, or air-proof conditions, are excluded, decomposition processes occur in a similar manner in whole bodies and dismembered body parts [16, 18, 45].

## Conclusion

This study shows that dismembered pig legs serve as suitable models for investigation of postmortem protein degradation and allow the establishment of the pig leg as a standardized model. This will help in various aspects, from the identification of external factors that influence post-mortem decomposition to the identification of new PMI markers. In a next step, we will use pig legs to precisely investigate to which degree postmortem degradation is influenced by ambient temperature, probably the most important external factor. Nevertheless, despite the evidence that degradation patterns of proteins are similar across mammalian species, the generalizability and applicability for human tissue is of primary interest for forensic routine and thus, there is no alternative to a validation of the present results by investigation of human degradation processes.

## Supplementary information

Fig. S1 Logistic regression curves of significantly PMI-correlated proteins represent the presence probability of certain protein products over the investigated time period. Regression curves are plotted within the PMI range from 0 to 240. With increasing PMI, the probability of all degradation events increases. In most cases, regression curves of amputated hind limb samples are steeper compared with attached hind limb samples and exceed the 95% confidence limit (*upper dotted horizontal line*) at lower PMI compared with non-amputated hind limb samples (PNG 1688 kb)

High Resolution (TIF 354 kb)

## Data Availability

All data generated or analyzed during this study are included in the published article.

## Abbreviations

GAPDH: glyceraldehyde-3-phosphate 12 dehydrogenase
hpm: hours postmortem
PMI: postmortem interval
