# Supplementary figures and images for: Dismembered porcine limbs as a proxy for postmortem muscle protein degradation
Source: Int J Legal Med. 2021 May 6;135(4):1627–36. doi: 10.1007/s00414-021-02571-6 (PMC8205874; doi:10.1007/s00414-021-02571-6)

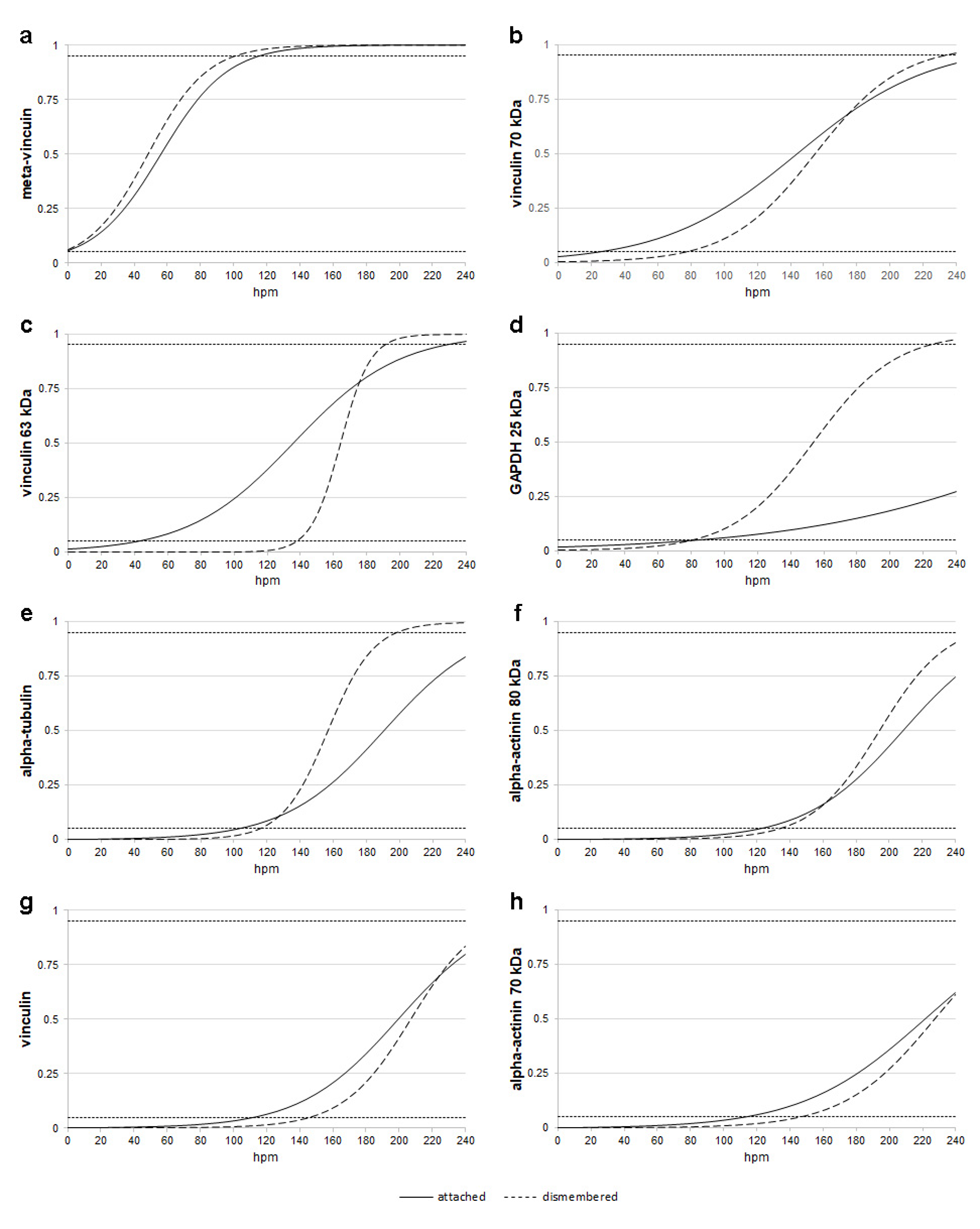

Supplement: Supplementary file 1 — Logistic regression curves of significantly PMI-correlated proteins represent the presence probability of certain protein products over the investigated time period. Regression curves are plotted within the PMI range from 0 to 240. With increasing PMI, the probability of all degradation events increases. In most cases, regression curves of amputated hind limb samples are steeper compared with attached hind limb samples and exceed the 95% confidence limit (upper dotted horizontal line) at lower PMI compared with non-amputated hind limb samples (PNG 1688 kb) [file 414_2021_2571_Fig4_ESM.png]

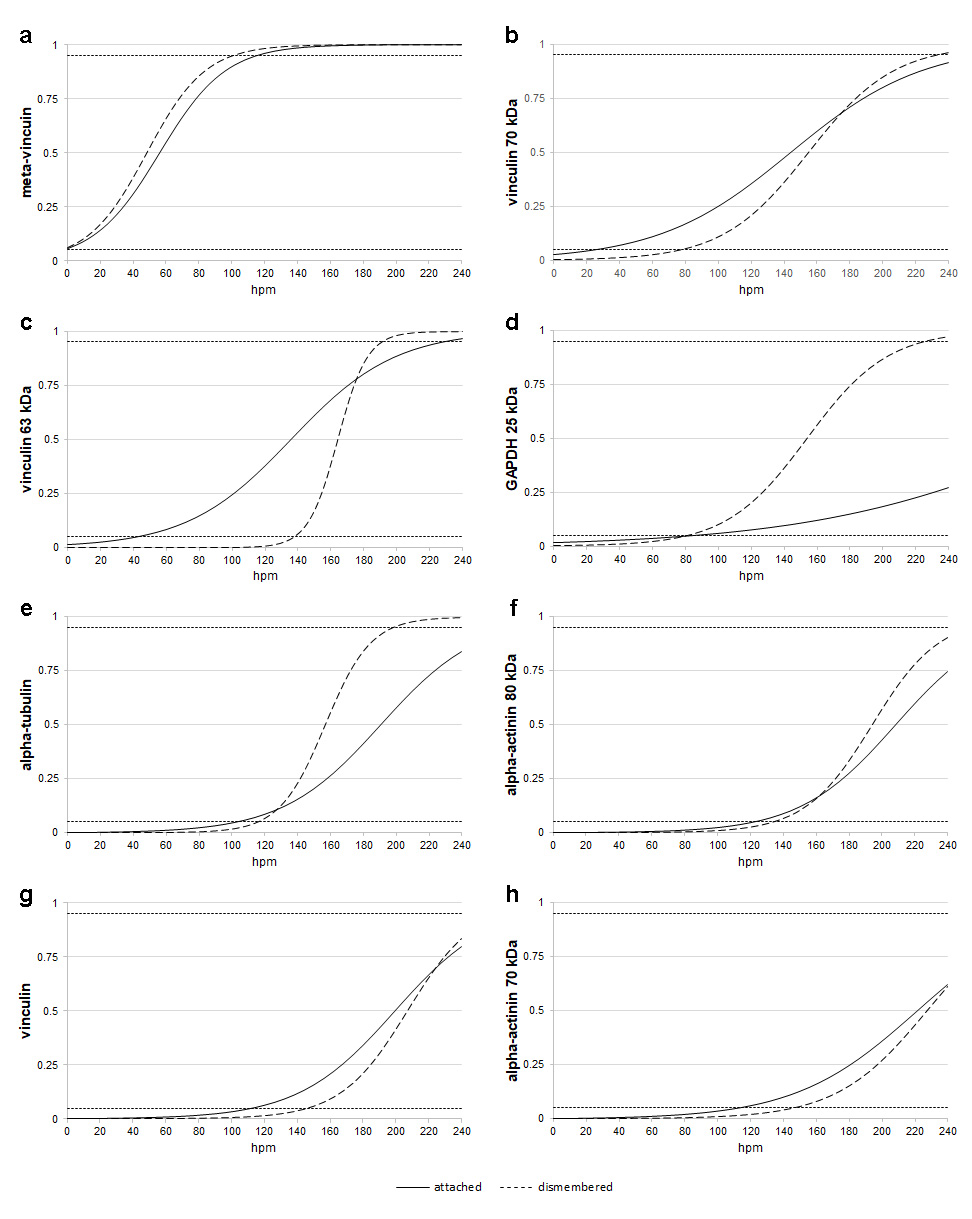

Supplement: Supplementary file 2 — High Resolution (TIF 354 kb) [file 414_2021_2571_MOESM1_ESM.tif]
